# Fractures in Osteogenesis Imperfecta: Pathogenesis, Treatment, Rehabilitation and Prevention

**DOI:** 10.3390/children9020268

**Published:** 2022-02-16

**Authors:** Wouter Nijhuis, Marjolein Verhoef, Christiaan van Bergen, Harrie Weinans, Ralph Sakkers

**Affiliations:** 1 Department of Orthopedic Surgery, University Medical Center Utrecht, 3508 GA Utrecht, The Netherlands; h.h.weinans@umcutrecht.nl (H.W.); r.sakkers@umcutrecht.nl (R.S.); 2 Department of Rehabilitation, Physical Therapy Science & Sports, Brain Center Rudolf Magnus, University Medical Centre Utrecht, 3508 GA Utrecht, The Netherlands; m.verhoef-10@umcutrecht.nl; 3 Department of Orthopedic Surgery, Amphia Hospital, 4818 CK Breda, The Netherlands; cvanbergen@amphia.nl; 4 Department of Biomechanical Engineering, Delft University of Technology, 2600 AA Delft, The Netherlands

**Keywords:** osteogenesis imperfecta, fracture, brittle bone disease, surgery, rehabilitation, collagen

## Abstract

Fractures in patients with osteogenesis imperfecta (OI) are caused by a decreased strength of bone due to a decreased quality and quantity of bone matrix and architecture. Mutations in the collagen type 1 encoding genes cause the altered formation of collagen type I, one of the principal building blocks of bone tissue. Due to the complexity of the disease and the high variation of the clinical problems between patients, treatment for these patients should be individually tailored. In general, short immobilization periods with flexible casting material, use of intramedullary implants, and simultaneous deformity correction are preferred. Multidisciplinary care with a broad view of the support needed for the patient and his/her living environment is necessary for the optimal rehabilitation of these patients. Increasing bone strength with exercise, medication, and sometimes alignment surgery is generally indicated to prevent fractures.

## 1. Introduction

Fractures are the main characteristic in patients with osteogenesis imperfecta (OI), also called “brittle bone disease”. OI is a genetic disorder with a disturbance of the production and structure of collagen type I, one of the main components of bone tissue. This rare bone disease has an incidence of 1 in 15,000–20,000 births [1]. The patients are generally clinically classified from type 1 to 5 [2], in which type 1 has the mildest symptoms and type 3 represents the most severe type compatible with life. Type 4 has a severity between type 1 and 3, and type 2 is defined as perinatal lethal. Type 5 has distinctive radial head luxation’s and ossification of the interosseous membrane. In addition to the Sillence classification, several rare types have now been described in the literature [3]. Eighty-five percent of the OI population has an autosomal dominant inheritance (types 1–5,15), of which the types 1–4 have a primary collagen type 1 defect. The remainder of 15% has an autosomal recessive inheritance, and the mutations in these patients affect the metabolic pathway of bone formation in different ways. A classification based on the metabolic pathway has been proposed by Forlino et al. in 2017 [3]. The list of OI types has been increasing; these additional types are clinically more or less similar to OI type 3 in terms of severity. Clinical manifestations vary widely between the different types of OI, ranging from patients who have mild symptoms with few fractures and a normal life expectancy, to patients with frequent fractures and severe bony deformities together with severe physical impairments and reduced life expectancy [4,5,6].

## 2. Collagen and Bone Tissue Formation

Bone tissue, or matrix, is made from collagen molecules and inorganic hydroxyapatite (HA) minerals. The structure of bone matrix can be compared with reinforced concrete, where the inorganic (HA) minerals are the cement, and the collagen molecules are the steel reinforcement. Biomechanically, the inorganic HA crystals give bone matrix its stiffness, and the reinforcement with collagen type 1 fibers creates the mechanical flexibility and related toughness. Bone tensile strength and resistance to both traction and shearing forces is mainly determined by the collagen network, making up around 30% of the bone matrix [7,8].

**Figure 1:**
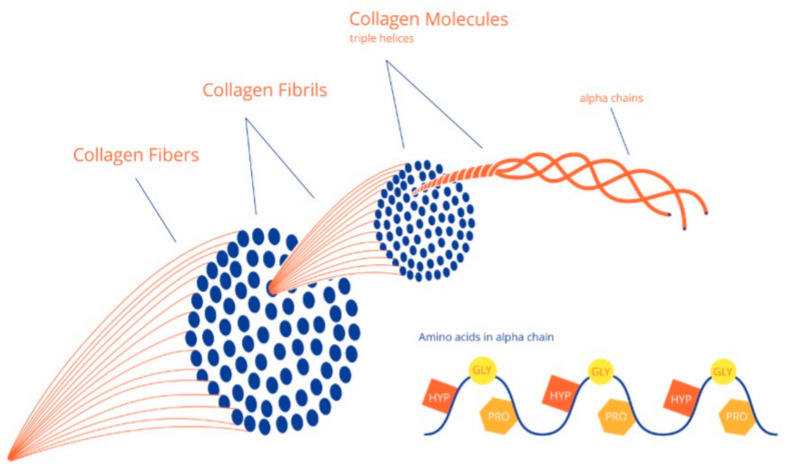
Multiple collagen fibrils form fibers. Reproduced with permission of Journal of Children’s Orthopedics [9].

**Figure 2:**
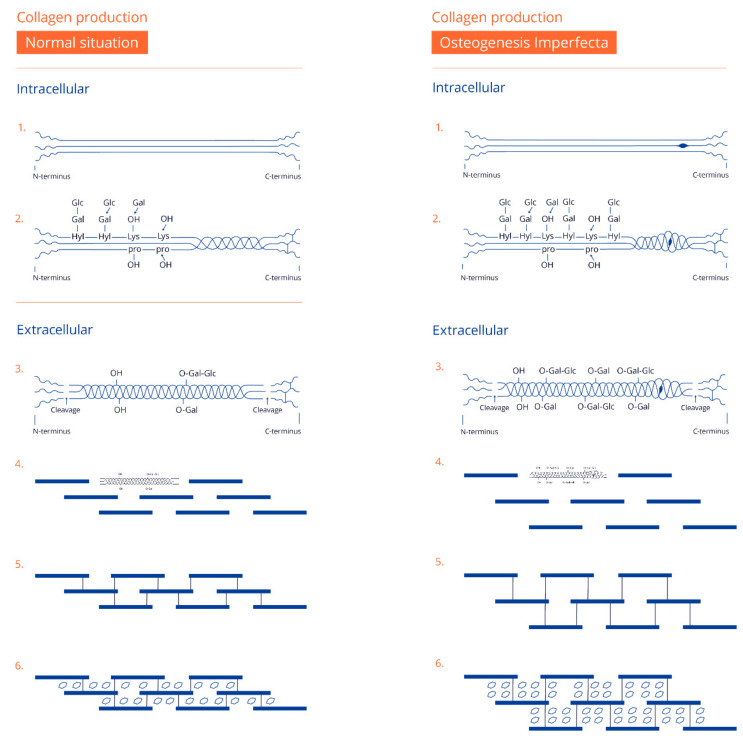
On the left side, a schematic view shows the formation of collagen, both intracellular and extracellular. On the right side, a similar formation is depicted, but with a mutation in one of the alpha chains, as is seen in osteogenesis imperfecta (OI). Step 1: formation of three alpha chains by ribosomes (note the bigger amino acid in one of the chains in OI). Step 2: hydroxylation and glycosylation and the triple helix formation (note the slower folding in OI with increased hydroxylation and glycosylation: Glucose (Glc), Galactose (Gal), Lysine (Lys), Hydroxylysine (Hyl), Proline (Pro)). Step 3: extracellular cleavage of the C- and N-terminus. Step 4: quarter-staggered arrays (note the increased space between the molecules in OI). Step 5: the formation of cross-links, which is unaffected in OI. Step 6: mineralization between the collagen molecules with an increased number of mineral crystals of the same size in OI. Reproduced with permission of Journal of Children’s Orthopaedics [9].

Collagen is made intracellularly and is a protein with a triple helix structure of peptide chains that are closely packed in a characteristic quarter-staggers array to form a fibril. A collagen fiber is made from multiple fibrils in the same way as multiple small iron threads together form a steel cable see Figure 1 and Figure 2 for more details on both normal collagen formation and collagen formation in OI [9].

After the extrusion of collagen by the cell into the extracellular matrix, inorganic HA is deposited as crystals in and between the fibrils and fibers, resulting in increased stiffening to finally become mineralized bone matrix (see Figure 2).

## 3. Bone Strength and Elasticity in OI

**Figure 3:**
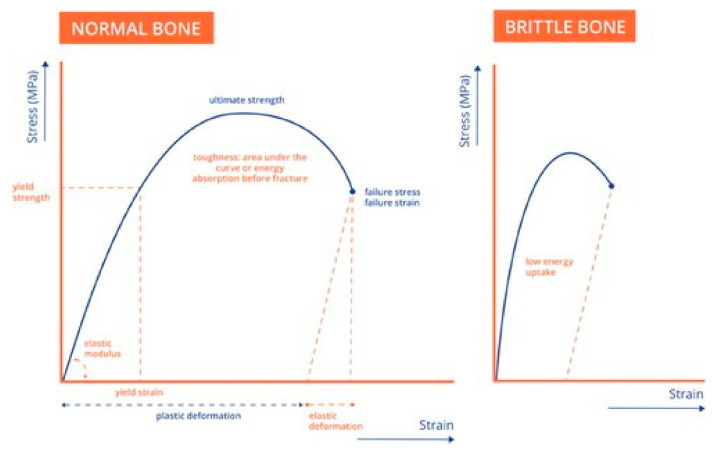
Brittle bone. Hypothetical stress-strain curve of bone with some of the most essential mechanical properties. For cortical bone, the deformations at yield are up to 1%, whereas for cancellous bone this can reach 5% to 10%, or even higher. Bone can absorb a substantial amount of energy and can be considered a relatively tough material (see area under the curve). Osteogenesis imperfecta bone is considered brittle, which means that it cannot absorb much energy (small area under the curve, right side). In fact, brittleness represents a combination of low strength, little plastic deformation, and lower toughness. Reproduced with permission of Journal of Children’s Orthopaedics [9].

The OI bone matrix has a lower capacity for energy absorption due to a lower strength and elasticity (Figure 3). The weaker bone matrix is susceptible to micro-damage, which causes increased activity of both osteoclasts and osteoblasts to repair these micro-damages. Increased osteoblast activity subsequently increases the osteocyte density, and thus leaves an increased porosity of bone due to osteocyte lacunae. Most likely, this increased activity at the cell level also causes an increased vascularity in the bone, which in turn adds on the increase in porosity. An increased pore percentage in OI bone has been reported at both the level of osteocyte lacunae and vascularity [10]. Next to the lower biomechanical properties of the bone matrix itself, a small rise in bone pore percentage might lead to significantly increased crack propagation through bone, in particular with repetitive loading and the accumulation of micro-damaged sites. For example, a bone pore percentage increase from 4% to 20% results in a three-fold decrease in the deformation abilities of bone before fracture [11], and thus much less energy uptake that refers to the bones brittleness (Figure 3).

Bone strength is also decreased at the level of bone micro and macro architecture. The refinement of bone imaging technologies in recent years has especially improved the assessment of bone architecture. Measures of bone micro-architecture, bone geometry, and (volumetric) bone mass density (vBMD) can be obtained by high-resolution peripheral quantitative computed tomography (HR-pQCT) [12]. A significantly decreased cortical thickness was found in tibiae of type 1 OI patients using HR-pQCT but normal to increased cortical thickness in OI types 3 and 4 [13]. Both histomorphometric and HR-pQCT evaluation of cancellous iliac bone biopsies in patients with OI showed fewer and thinner trabeculae [13,14,15] The Trabecular Bone Score (TBS) as measured with HR-pQCT is related to trabecular connectivity and trabecular spacing, and low TBS in peripheral bone has a strong association with individual fracture risk [16]. In OI patients type 3 and type 4, lower TBS values were found compared to normal bone [13]. All these pathologic changes at the different architectural levels also add to the susceptibility of fractures in patients with OI.

## 4. Fracture Management

Fracture management in OI patients need a very tailor-made approaches, but general principles apply to all OI patients. When OI patients suffer from a fracture, emergency treatment should preferably be provided at the nearest possible institution. While some fractures do not require any treatment, some fractures need immobilization with plaster of Paris (POP), and some fractures need surgery with reinforcing intramedullary implants. Patients and family education on OI can be very beneficial in pain management, transportation, and logistics in these emergencies. Some clinics teach patients and/or parents to apply a temporary plaster or splint themselves in order to cope with the first pain before adequate professional healthcare is available. When POP is utilized, we prefer the use of flexible material over a rigid cast. Since the bone in OI is brittle, the end of a rigid cast might create a stress riser, which increases the risk of additional fractures during treatment with a cast. This risk could be minimized with more flexible cast materials.

The average time to consolidation in OI patients is lower than in controls, due to a higher bone turnover in OI bone (higher vascularity and higher cellularity). Hence, a relatively short period of immobilization is often sufficient. When surgical treatment is indicated, the authors feel that pre-existing deformity of the bone should be repaired where possible to minimize the risk of re-fracture. The simultaneous correction of deformities on the contralateral side and adjacent bone should also be considered in order to minimize frequency of surgeries and periods of immobilization for all OI patients.

## 5. Prevention of Fractures

### 5.1. Medication

As for all children, maintaining adequate vitamin D (vit D) concentrations is one of the basic pre-requisites for normal bone mineralization and bone mass [17]. Children with OI seem to be at risk for vit D deficiency, especially those with more severe OI and/or a high body mass index [18]. Therefore, children with OI should have their vit D status monitored and be supplied with a dietary vitamin D supplement to ensure optimal levels [9]. Bisphosphonate (BP) treatment is now widely employed in OI patients to improve bone mass. BP decreases bone resorption by osteoclasts, shifting the balance of bone resorption and bone formation towards more OI bone formation [19]. Based on bone mineral density (BMD) and fracture rate, BP treatment might be started early. A low BMD is not the only factor causing lower bone strength in OI. The porosity, architecture, and connectivity within bone structure, as well as the quality of collagen fibers and the collagen to mineral ratio, play important roles in decreased bone strength. BP treatment can decrease pore percentage and increase BMD, but cannot change all factors involved in the decreased bone strength in OI.

Although the impact is not completely understood yet, BPs affect osteoblast and osteocyte activity directly. The main effect of BPs in the treatment of OI lies in the modulation of osteoclast activity, altering the structure and the architecture of bone [9]. A decrease in bone turn-over is usually not a problem in children. Children with OI have a higher bone turn-over compared to children without OI. Since adults have a decrease in bone turn-over with increasing age, bisphosphonates should be used with caution in adult OI patients. Indications are stricter, and lower frequencies of BP admission is advised [20] in order to keep turnover at a minimum level, which is a prerequisite to prevent the accumulation of micro-damage and related so-called ‘spontaneous’ fractures [21].

Increased BMD is usually most prominent in the first year of treatment. The effect of BP is patient specific and should be monitored yearly with fracture frequency and BMD. Since BP therapy only modulates osteoclast activity, some researchers focus on new medication strategies, a phase 1 study on mesenchymal stem cell treatment [22] in which they try to create a mosaic DNA is ongoing. A phase 2a clinical trial with antisclerostin [23] together EMA and FDA approval opens the path to a phase 3 pediatric study with antisclerostin in OI. Antisclerostin increases osteoblast activity rather than decreasing osteoclast activity, as BP do. Results from this research might change the outcomes for patients with OI in the near future.

### 5.2. Physical Activity

Good motor skills and physical activity are important for proper bone development and the prevention of fractures in all people. During exercise, the effect of loading has positive influences on both the quantity and quality of bone [24]. In OI, collagen type 1 affects not only the bone, but also the soft tissues in the musculoskeletal system. Increased laxity, quantified by using the Beighton score, is often found in these patients. Furthermore, muscle weakness is present more often. Muscle weakness might be directly related to the altered collagen type 1 formation [25]. Keeping the patient physically active with tailor-made activity training programs is mandatory.

### 5.3. Preventive Surgery

**Figure 4:**
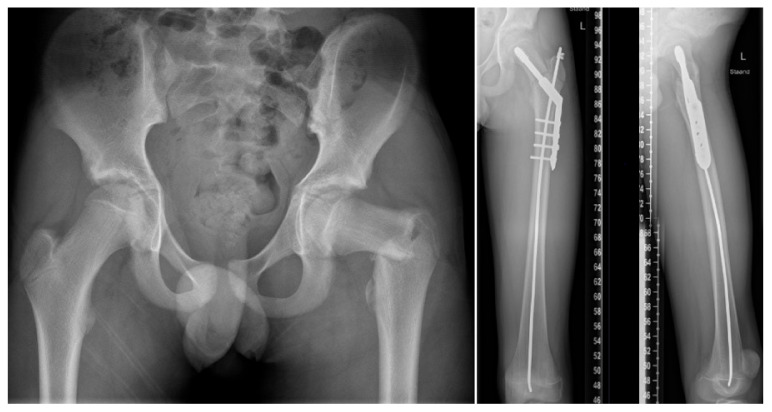
Adolescent patient with OI type 4 and a proximal femur fracture, treated with a dynamic hip screw and long intramedullary nail to reduce stress rising at the end of the plate. Using a monocortical distal screw would have further reduced stress rising. Note the stopper on the nail instead of a proximal bend end to prevent migration of the rod into the intramedullary canal.

Alignment surgery with intramedullary rodding to increase bone strength should be considered to decrease the fracture risk in children with moderate to severe OI. Teamwork and clear communication with the patient and family, surgeon, and multidisciplinary team are essential for a shared decision-making process. Pre-operatively, baseline function, range of motion, muscle strength and length, pain, and quality of life should be measured, preferably using standardized and validated outcome measures [26], and re-assessed after fracture treatment. Elongating implants or constructs for stable longitudinal growth are usually preferred in growing children. The inserted intramedullary rods are left in the bones as long as possible to prevent re-fractures. However, the re-operation rate due to rod migration and telescoping failure is very high, and re-revision rates of 30% within 5 years of follow-up have been reported with the current elongating devices [27]. Fixed-length devices can be used as an alternative when bone size is small, for children with limited residual growth or when lengthening devices are not available [28]. Plates and screws as stand-alone implants should be avoided to prevent stress fractures at the edges of the plates (see Figure 4) in all OI patients.

Postoperative immobilization is provided with a flexible backslab or plaster cast, followed by initiation of mobilization as soon as healing permits [28].

General considerations and recommendations about surgical treatment in both fracture management and preventive surgery for both children and adults have recently been published after an international task force reached consensus in a standardized way [28].

**Figure 5:**
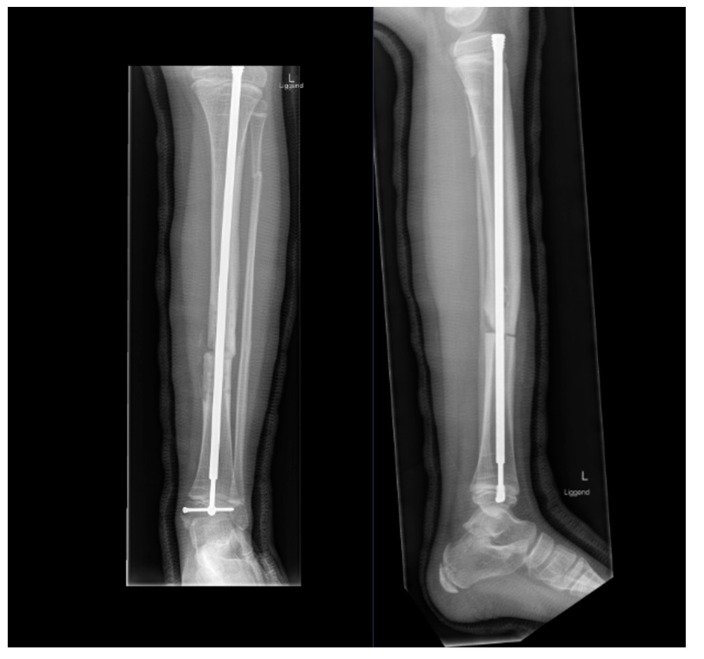
Eight-year-old OI type 1 patient with a delayed diagnosis presented with the fourth fracture of the tibia before OI was diagnosed. The fractures were treated conservatively and with a stand-alone plating. After removal of the last plate a third re-fracture of the tibia occurred within 1 week. To prevent any further re-fractures, a correction osteotomy at the site of the fracture was performed and stabilized with an elongating device. Note the improved distal fixation with a small screw (Peg).

**Figure 6:**
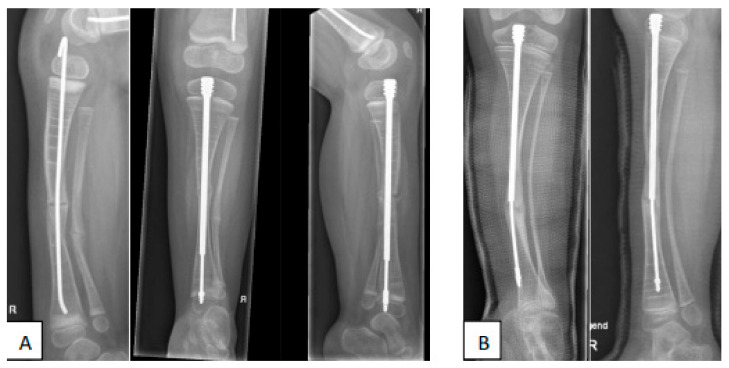
**(A)**: Four-year-old OI type 3 patient after correction osteotomy of the tibia, stabilized with a flexible intramedullary nail, which was complicated with proximal nail migration causing knee pain. This complication was solved by replacing the nail with an intramedullary elongating device. **(B)**: Three years later, valgus deformation had occurred, distal fixation was lost, and the device bent, migrating out of the intramedullary canal distally.

Figure 4, Figure 5 and Figure 6 show some illustrative cases with different surgical techniques and failures.

### 5.4. Rehabilitation

After fracture management or elective surgery, patients need a personalized rehabilitation program. An early start of rehabilitation after surgery is strongly advised. The rehabilitation program should preferably be done in the patient’s own environment. A multidisciplinary OI team should provide a rehabilitation program and stay connected to both patient and their local health care professionals during the rehabilitation. If the patient will wear a cast or have a partial on non-weightbearing regime, patients and their families need to learn safe methods for transfers and daily care before discharge from hospital. It is advisable to practice these transfers before surgery to become familiar with the available aids and prescribed methods. The rehabilitation program should focus on range of motion, muscle strength, and improvement of general functioning. Besides that, psychological support can be beneficial for some patients. Any rehabilitation progress should be evaluated in line with patient and family goals and surgeon’s protocol.

In addition to rehabilitation care following the acute phase of fractures or surgery, continuous guidance from a rehabilitation team is important to support children with OI in optimal development and participation in society (e.g., mobility, personal care, school, and sports). The international classification of functioning, disability, and health (ICF) can be used as a framework to cover all important domains. Recently, an international interdisciplinary working group called Key4OI developed a set of global outcome measures for patients with OI using a consensus-driven modified Delphi approach. The Key4OI screening set is recommended for regular screening of daily functioning and quality of life [26].

## 6. Multidisciplinary Treatment Challenges

There is no cure for OI yet. Current treatment is based on increasing bone mass, prevention of fractures with alignment surgery, fracture management, and rehabilitation. The main treatment goal for children with OI is optimizing mobility, functional independency, and participation in society. The high variability in clinical severity of OI makes standard care recommendations less appropriate. Individually tailored care should therefore be the standard for OI patients. Children with OI as well as their parents often develop a fear of fractures. Health care professionals not used to treating OI patients have this fear as well. This may keep the patient from reaching their full potential in functioning. Therefore, caregivers should be educated how to handle a child with OI from birth [29]. Overprotection by both patients, their family members, and local healthcare providers may lead to a vicious cycle of fracture, immobilization, deconditioning, reduced skeletal strength and re-fracture. This needs to be addressed in a tailor-made rehabilitation program, including a psychological approach [30,31]. All treatment modalities should focus on optimal functioning of the child and family. A team with support of social workers and psychologists might be indicated.

## 7. Conclusions and Future Perspectives

Next to providing fracture management and prevention in patients with OI, multidisciplinary care should focus on functioning and psychosocial well-being. Despite all research and advances, the current management of fractures in OI remains a combination of surgery and medical treatment and requires a tailor-made approach from a multidisciplinary team of OI experts from different specialties.
